# Antigen-Specific Immunotherapy with Thyrotropin Receptor Peptides in Graves' Hyperthyroidism: A Phase I Study

**DOI:** 10.1089/thy.2019.0036

**Published:** 2019-07-17

**Authors:** Simon H.S. Pearce, Colin Dayan, David C. Wraith, Kevin Barrell, Natalie Olive, Lotta Jansson, Terrie Walker-Smith, Christina Carnegie, Keith F. Martin, Kristien Boelaert, Jackie Gilbert, Claire E. Higham, Ilaria Muller, Robert D. Murray, Petros Perros, Salman Razvi, Bijay Vaidya, Florian Wernig, George J. Kahaly

**Affiliations:** ^1^ Institute for Genetic Medicine, Newcastle University, and Newcastle Hospitals NHS Trust, Newcastle upon Tyne, United Kingdom.; ^2^ Thyroid Research Group, School of Medicine, Cardiff University, Cardiff, United Kingdom.; ^3^ Institute of Immunology and Immunotherapy, University of Birmingham, Birmingham United Kingdom.; ^4^ Apitope Technology (Bristol) Ltd., Chepstow, United Kingdom.; ^5^ Apitope International NV, Diepenbeek, Belgium.; ^6^ Institute of Metabolism and Systems Research, University of Birmingham, Birmingham United Kingdom.; ^7^ Department of Endocrinology, King's College Hospital, London, United Kingdom.; ^8^ Department of Endocrinology, Christie Hospital NHS Foundation Trust, University of Manchester, Manchester Academic Health Science Centre, Manchester, United Kingdom.; ^9^ Department of Endocrinology, St. James's University Hospital, Leeds, United Kingdom.; ^10^ Endocrine Unit, Newcastle Hospitals NHS Trust, Newcastle upon Tyne, United Kingdom.; ^11^ Institute for Genetic Medicine, Newcastle University, Newcastle upon Tyne, United Kingdom.; ^12^ Macleod Diabetes & Endocrine Centre, Royal Devon and Exeter Hospital, Exeter, United Kingdom.; ^13^ Department of Endocrinology, Imperial College, London, United Kingdom.; ^14^ Department of Medicine I, Johannes Gutenberg University Medical Center, Mainz, Germany.

**Keywords:** peptide immunotherapy, Graves' disease, autoimmune thyroid disease, desensitization, thyroid stimulating hormone receptor, immunomodulation

## Abstract

***Background:*** Graves' disease is one of the most common autoimmune conditions, but treatment remains imperfect. This study explores the first-in-human use of antigen-specific immunotherapy with a combination of two thyrotropin receptor (TSHR) peptides (termed ATX-GD-59) in Graves' hyperthyroidism.

***Methods:*** Twelve participants (11 female) with previously untreated mild to moderate Graves' hyperthyroidism were enrolled in a Phase I open label trial to receive 10 doses of ATX-GD-59 administered intradermally over an 18-week period. Adverse events, tolerability, changes in serum free thyroid hormones, and TSHR autoantibodies were measured.

***Results:*** Ten subjects received all 10 doses of ATX-GD-59, five (50%) of whom had free triiodothyronine within the reference interval by the 18-week visit. Two further subjects had improved free thyroid hormones by the end of the study (7/10 responders), whereas three subjects showed worsening thyrotoxicosis during the study. Serum TSHR autoantibody concentrations reduced during the study and correlated with changes in free thyroid hormones (*r* = 0.85, *p* = 0.002 for TSHR autoantibody vs. free triiodothyronine). Mild injection-site swelling and pain were the most common adverse events.

***Conclusions:*** These preliminary data suggest that ATX-GD-59 is a safe and well-tolerated treatment. The improvement in free thyroid hormones in 70% of subjects receiving the medication suggests potential efficacy as a novel treatment for Graves' hyperthyroidism.

## Introduction

Graves' disease (GD) is the most common cause of hyperthyroidism, and results from the production of autoantibodies that stimulate the cell-surface thyrotropin receptor (TSHR) (1). It affects around 3% of women and 0.5% of men over a lifetime, and most commonly presents in the fourth and fifth decades of life, with a disproportionate burden of ill health falling on working-age women (2).

There have been no new treatment options for hyperthyroid patients with GD for >60 years (3). At present, antithyroid drugs (ATD), mostly methimazole and its pro-drug carbimazole, are the usual first-line treatment, with radioactive iodine and total thyroidectomy reserved as second-line therapies in most health-care environments (4). Each of these therapeutic modalities has significant drawbacks, in particular that ATDs induce drug-free remission in only 50% of people (5), and that both radioiodine and thyroidectomy result in lifelong hypothyroidism. In addition, there is a small risk of agranulocytosis with ATDs, occurring in around 0.2–0.5% of patients (6,7). Many GD patients are also apprehensive about the theoretical hazards of radioiodine administration, and young patients are frequently keen to avoid the risk of surgery and the anterior neck scar that results from conventional thyroidectomy. These factors mean that there is currently no ideal treatment for GD, and that each patient makes a trade-off between the certainty of cure and the different undesirable effects with each therapy (8).

Antigen-specific immunotherapy, administering small amounts of antigens over time, often reinstates immune tolerance in allergy (9), but in autoimmune diseases, the approach has been associated with complications from immune responses to the administered antigens (10–12). Over recent years, it has been shown that the risk of an immune response can be minimized during tolerance induction by the use of synthetic peptides that mimic naturally processed CD4+ T-cell epitopes (13,14). Such peptides have been termed “apitopes,” short for antigen-processing independent epitopes (15). Apitopes have successfully induced self-tolerance in multiple sclerosis (16), and the mixture of myelin basic protein (MBP) peptides used (ATX-MS-1467) is currently in Phase IIb development. A similar approach has also shown preliminary evidence of efficacy in type 1 diabetes (17).

ATX-GD-59 is an admixture of two soluble, synthetic peptides based on the sequence of the human TSHR that have been shown to function as apitopes in HLA-DR3 transgenic mice immunized with human TSHR (18). One peptide, 9B-N (15 amino-acid residues), is an exact sequence found in TSHR, whereas the other, 5D-K1 (21 amino-acid residues), contains an amino-acid sequence found in the TSHR with the addition and replacement of charged amino acids to the N- and C-terminal flanks for increased solubility. Both peptides overlap with residues previously documented as TSHR immunodominant T-cell epitopes in HLA-transgenic murine models of GD (19,20). ATX-GD-59 is thought to bind with high affinity to HLA-DR molecules on the surface of immature dendritic cells in the draining lymph node without prior internalization, thereby reducing the risk for activation of antigen-presenting cells (18).

Here, the results from an open-label Phase I study to assess the safety and biological activity of ATX-GD-59 in adult subjects with Graves' hyperthyroidism not currently treated with antithyroid therapy are reported for the first time.

## Methods

### Study design

This was an open-label study conducted at eight National Health Service endocrinology centers in the United Kingdom. The primary objective was to assess the safety and tolerability of intradermal (i.d.) administration of ATX-GD-59 in subjects with GD not currently treated with ATDs. Secondary outcomes were to explore biological activity, including changes in hormonal, immunological, and TSHR antibody levels, with treatment. The study was designed in full compliance with the ethical requirements set out in the Declaration of Helsinki (21) and the International Conference on Harmonisation (ICH), as well as with local regulations (22). All subjects gave their written informed consent to participate. The trial was registered with clinicaltrials.gov (NCT02973802) and approved by the East of England (Essex), Research Ethics Service (16/EE/0133). The full study protocol is available at www.daviad.eu

Eligible subjects were between 18 and 65 years of age with mild to moderately severe GD diagnosed by a physician from clinical and laboratory findings, and none had received prior treatment with ATD. Subjects had to carry at least one major histocompatibility complex (MHC) class II HLA-DRB1*15, HLA DRB1*03, or HLA DRB1*04 allele, and have quantifiable levels of TSHR antibodies, elevated levels of free triiodothyronine (fT3) and/or free thyroxine (fT4; not exceeding 15 pmol/L and 35 pmol/L, respectively), and undetectable levels of thyrotropin (TSH) at screening. These MHC allelotypes have been demonstrated to bind the administered peptides (19). Men who were not sterile and women of child-bearing potential had to commit to birth-control measures. Main exclusion criteria were pregnancy or breastfeeding in women, treatment with any ATD, steroids, or adrenocorticotropic hormone (with the exception of inhaled steroids), or cytokine or anti-cytokine therapy within the previous three months prior to study day 1, previous treatment with biological or peptide-based immunotherapy, previous treatment with radioiodine, partial or complete thyroidectomy, or detectable levels of antibodies in plasma specific for any of the peptides within ATX-GD-59. Treatment with oral propranolol was permitted throughout the study.

**FIG. 1.**
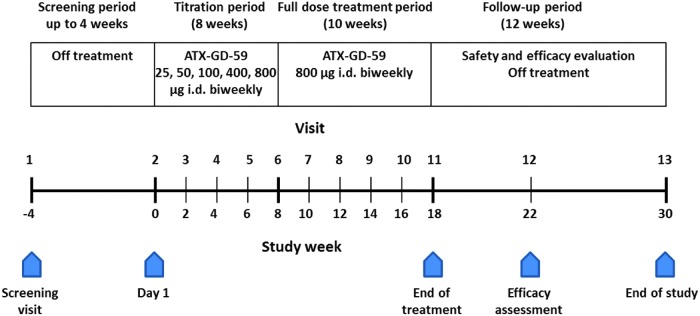
Time course, titration scheme, and visit schedule during the study.

This was an open-label study in which all participants received ATX-GD-59. The time course, titration schedule, and visits are shown in Figure 1. ATX-GD-59 is a lyophilized equimolar mixture of two peptides (peptide 5D-K1: KKKKYVSIDVTLQQLESHKKK, and peptide 9B-N: GLKMFPDLTKVYSTD) reconstituted at the clinic to 4 mg/mL and 0.5 mg/mL total peptide content, depending on dose, prior to i.d. injection. The starting dose was 25 μg i.d., and the maximum single dose was 800 μg, based on other immunotherapy studies with apitopes (15,16,18), and included a safety margin compared to doses used in repeat-dose toxicity studies in a rodent model.

The first four subjects were recruited sequentially with a minimum of 48 hours between the first dose of each subject. Individual subject safety data were reviewed by the investigator and the medical monitor after the first dose of ATX-GD-59. Dosing of the remaining subjects only commenced once the first subject has received the second dose without safety concerns identified.

During an eight-week period, the dose was titrated biweekly up to a final dose of 800 μg. Subjects were then maintained on the full dose for 10 weeks, followed by a 12-week safety and evaluation period off treatment.

### Outcome measures

Data were gathered by site investigators and entered into electronic case report forms for analysis. The primary outcome measure was the occurrence of treatment-emergent adverse events (TEAE), serious AE including treatment-emergent serious adverse events (TESAE), and laboratory abnormalities assessed on an ongoing basis up to week 22. Secondary outcomes measures were serum fT3, fT4, and TSH concentrations assayed at a central site (Roche, Centaur platform; Quintiles/IQVIA, Livingston, United Kingdom). Patients whose circulating thyroid hormone concentrations (fT4 or fT3) reduced during the dosing period compared to baseline measurements were designated as responders. Changes in TSHR antibody levels measured by both a TSHR antibody binding-inhibition assay (TBII; Cobas; Roche, Basel, Switzerland) and by *in vitro* stimulation of a Chinese hamster ovary cell line expressing the TSHR (TSHR-stimulating antibodies [TSAb] cell-based bioassay; Thyretain, Quidel, San Diego, CA), as described (23), were also exploratory secondary outcomes. The TSHR antibody measurements were done at Johannes Gutenberg University Medical Center (Mainz, Germany) (23).

### Documentation of AEs and concomitant medications

All AEs were recorded at each clinic visit and characterized by severity and relationship to the study drug according to Medical Dictionary for Regulatory Activities (MedDRA 19.1) terminology. The indication for the concomitant medications was recorded along with the dose and the period of therapy. During the dosing phase, subjects were observed in the clinic for at least two hours after administration of the study drug with regular monitoring of vital signs. Laboratory hematology, clinical chemistry, and urinalysis safety measurements were performed at all visits.

### Statistical methods

Data are presented descriptively. Baseline, safety, and efficacy data are presented for the primary intention to treat (ITT) population, comprising all subjects who received one or more doses of study drug. Means ± standard deviation and medians (min–max) are presented as appropriate; changes from baseline are given as percentages. Correlations between change in thyroid function tests and TRAb levels were analyzed using the parametric Pearson's test. Outcome data were analyzed for all participants who received the full 10 doses of ATX-GD-59. For this Phase I study, no formal sample size calculation was performed.

## Results

### Participant flow

**FIG. 2.**
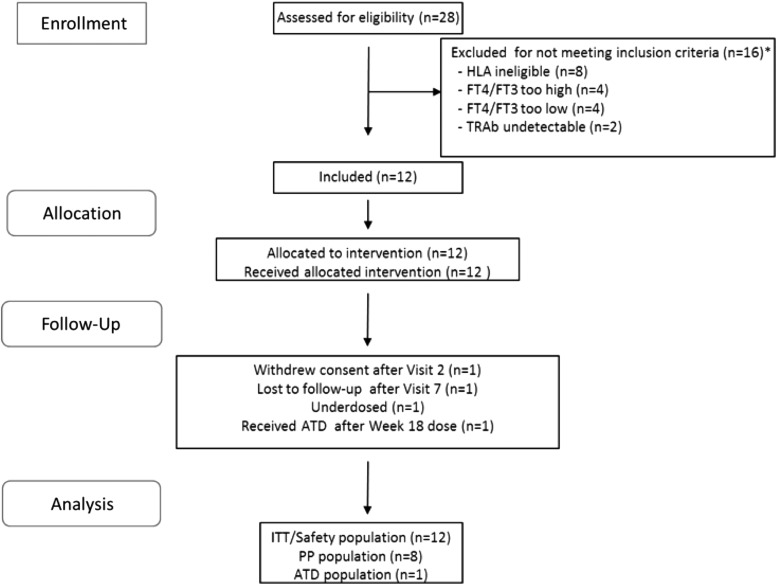
Patient flow (CONSORT). The asterisk indicates two screened subjects who were excluded on two counts: HLA ineligible and fT4/fT3 values too high (*n* = 1) or too low (*n* = 1). ITT, intention to treat, PP, per protocol, ATD, antithyroid drug; fT4, free thyroxine; fT3, free triiodothyronine.

Out of the 28 subjects screened, 12 were enrolled in the study and received treatment. Due to enrolment time constraints, the estimated protocol target of 15 subjects was not achieved. The flow of participants is shown in Figure 2. The safety (ITT) population comprised 12 subjects who received at least one dose of the study drug. Ten subjects completed the study, and of these, eight received the treatment regimen in full compliance with the protocol. Two subjects did not complete the study, as one was lost to follow-up and the other withdrew consent for non-safety reasons. One subject took ATDs on the day of but after the final dose (at week 18). One subject was unintentionally underdosed throughout the study. One subject had elevated fT3 and fT4 concentrations at the screening visit and was enrolled per protocol (PP), but the concentrations were found to have returned to the reference range following dosing at visit 1 (her TBII antibodies were positive). One subject had asymmetrical upper eyelid retraction at baseline, but none had active Graves' orbitopathy. Demographic data of the safety population are shown in Table 1. Of the 10 subjects who completed the full study dose regimen, six received oral propranolol (40–160 mg daily) during the study: three from before the first study visit and three subsequently (at weeks 2, 4, and 7).

**Table 1.** Demographic Characteristics of the Study Population at Screening (ITT Population, *n* = 12)

*Variable*	*Value*
Age (years), *M* ± *SD*	42.7 ± 9.7
Female sex, *n* (%)	11 (92%)
BMI (kg/m^2^), *M* ± *SD*	28.0 ± 5.1
Family history of Graves' disease at screening, *n* (%)	2 (17%)
fT3 (pmol/L), median (min–max), ref: 3.5–6.5	9.3 (6.8–13.4)
fT4 (pmol/L), median (min–max), ref: 11.5–22.7	24.2 (17.7–35.0)
TSH (mIU/L), median (min–max), ref: 0.4–4.0	0.01 (0.01–0.02)
HLA typing (allele 1 and 2)	
DRB1^*^03:01	8 (67%)
DRB1^*^04:0X	6 (50%)
DRB1^*^15:01	3 (25%)
Non-DRB1^*^03,^*^04, or ^*^15	7 (58%)
Pre-dose TBII (IU/L), median (min–max], cutoff: <1.75 IU/L	3.77 (1.84–15.66)
Pre-dose TSAb (%), median (min–max), cut-off: SRR% <140%	406.0 (234–1052)

HLA DRB1^*^04:0X alleles include alleles ^*^04:01 (*n* = 1), ^*^04:02 (*n* = 3), ^*^04:04 (*n* = 1), and ^*^04:07 (*n* = 1). NB: To be eligible, only one suitable HLA allele was required.

TSHR, thyrotropin receptor; TBII, TSHR-binding inhibitory immunoglobulin; TSAb, stimulatory TSHR antibodies; SRR, specimen-to-reference ratio.

### Safety results

A total of 11 subjects reported 311 TEAEs during the study, of which 180 were considered treatment related. A summary is provided in Table 2. Of the 311 TEAEs, 293 events were mild, and 18 events were moderate in intensity. No severe AEs or deaths were reported. The majority of treatment-related TEAEs were injection-site reactions, the most common reported as erythema, swelling, and pain, which accounted for 153 of all 180 treatment-related TEAEs, and all were mild in severity. No TEAEs leading to drug interruption, suspension, or early withdrawal were reported. The remainder of the TEAEs included various inconsistent minor conditions, including headache, nausea, food poisoning, upper respiratory tract, skin, and fungal nail infections. There was no discernible difference between the frequency of study drug–related AEs between the titration period and the full-dose treatment period (Table 2).

**Table 2.** Most Frequent TEAEs

	*Number of subjects (number of events)*	
*Variable*	*Titration period overall (*N* = 12)*	*Full treatment period, 800 μg (*N* = 11)*
TEAEs	11 (157)	10 (154)
Drug-related TEAEs	11 (93)	10 (87)
TESAEs	1 (1)	1 (2)
Drug-related TESAEs	1 (1)	0
TEAEs leading to death	0	0
TEAEs leading to early study withdrawal	0	0
TEAEs leading to dose interruption or suspension	0	0
Treatment-emergent intradermal injection–related reaction	11 (74)	10 (79)
TEAEs by maximum severity:		
Mild	11 (150)	10 (143)
Moderate	5 (7)	5 (11)
Severe	0	0

TEAEs, treatment-emergent adverse events; TESAEs, treatment-emergent serious adverse events.

A single subject experienced three TESAEs on different occasions: nausea (moderate intensity, possibly related to drug), vomiting (mild intensity, not related to drug), and atrial fibrillation (mild intensity, not related to drug). For each of these AEs, the categorization as serious was based on new or prolonged hospitalization for observation. Another subject developed first-degree heart block at week 4, which improved when concomitant propranolol was discontinued. One subject tested positive for antidrug antibodies at week 14 and remained positive at week 22. There were no accompanying clinical sequelae.

### Efficacy results

**FIG. 3.**
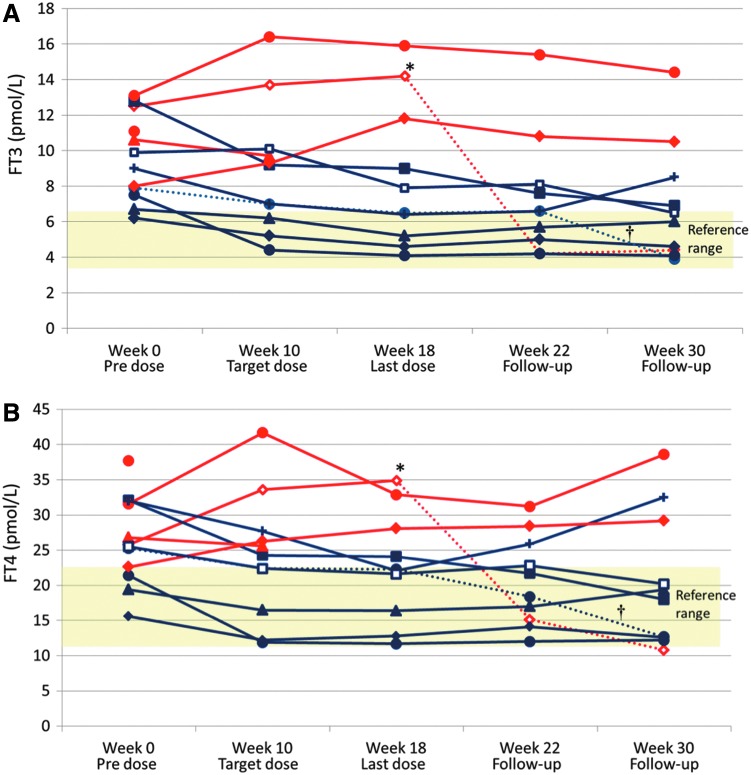
Individual levels of fT3 (**A**) and fT4 (**B**) over time in the ITT population. Blue lines represent responders, defined as those who showed a reduction in levels of both fT3 and fT4 at week 18. The dotted blue line indicated with a dagger represents one subject who was underdosed. This subject also received ATDs after week 22. Red lines represent non-responders. The dotted red line indicated by an asterisk represents one subject who received ATDs after week 18. Fields shaded yellow indicate reference ranges.

The absolute levels of serum fT3 and fT4 for each subject are shown in Figure 3. Two subjects did not complete the full dosing schedule, discontinuing treatment following one and six doses. Of 10 participants completing follow-up, five (50%) had fT3 within the reference range following the 10 doses of ATX-GD-59, including one subject who was underdosed with the study medication; a further two subjects had lower serum fT3 and fT4 concentrations at 18 weeks than at baseline (making 7/10 subjects with a total or partial response). Two participants had detectable serum TSH at week 18—one 0.11 mIU/L and the other 2.56 mIU/L—with the remaining subjects' TSH concentrations remaining at ≤0.02 mIU/L. Three subjects had small but progressive elevations in fT3 and fT4 over the same time period. No subject required intervention with ATDs during the 18-week dosing period, but one subject received carbimazole following the final dose of ATX-GD-59 at week 18 and a second following the week 22 assessment (shown as dashed lines in Fig. 3). Two of the three non-responders and four of seven responders took propranolol during the active treatment phase. Two patients remained euthyroid without ATDs for a year after their last dose of peptides; one became transiently hypothyroid.

**FIG. 4.**
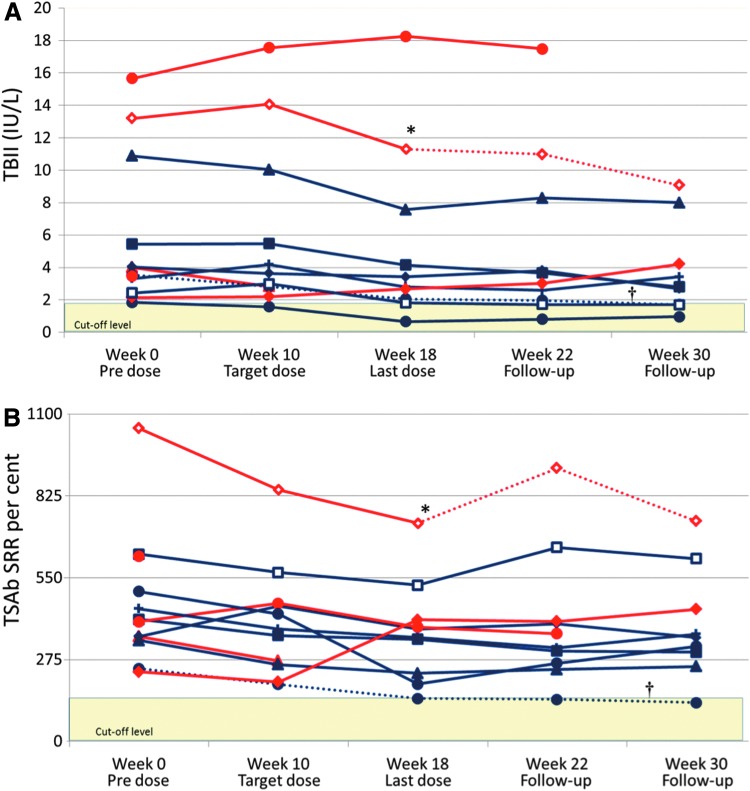
Individual levels of thyrotropin receptor (TSHR) antibodies, measured as (**A**) TSHR binding inhibitory immunoglobulin (TBII) and (**B**) TSHR stimulatory antibodies (TSAb). Blue lines represent responders. The dotted blue line indicated with a dagger represents one subject who was underdosed. This subject also received ATD after week 22. Red lines represent non-responders. The dotted red line indicated by an asterisk represents one subject who received ATDs after week 18. Thresholds for positive antibody concentrations are 1.75 IU/L for TBII and 140% for TSAb, shown as yellow-shaded fields.

Overall, there were reductions in both TBII and TSAb concentrations across the participant cohort over the study period, but they remained elevated in most subjects (Fig. 4). There were significant correlations between changes in serum fT3 concentration and changes in both TBII and TSAb from baseline to week 18 (*p* = 0.002 and *p* = 0.02, respectively; see Supplementary Fig. S1). Reduction in serum fT4 was also associated with falling TBII levels (*p* = 0.03), with a similar trend toward association with TSAb concentration (*p* = 0.11).

## Discussion

Hyperthyroidism in GD presents as a consequence of the direct effects of stimulating TSHR autoantibodies on thyroid function, rather than because of prolonged tissue damage or inflammation. Thus, unlike type 1 diabetes or Addison's disease, where it is estimated that 80–90% of the relevant functional endocrine tissue has already been destroyed before symptoms of hormonal deficiency occur (24,25), GD may present more quickly in relation to the evolving immune response. This means that GD may be particularly amenable to immunotherapeutic approaches, as the immune response may be less well established in GD than in other autoimmune conditions. Furthermore, the underlying hormone-producing tissue is not destroyed, leaving the opportunity for normal function in the future if the immune response can be modified or abrogated.

The extracellular domain (ECD) of the TSHR is very well characterized as the target of both the humoral and cell-mediated autoimmune responses in GD (26), and preclinical work on small-molecule “drug-like” ligands acting as TSHR agonists has helped yield important insights in the pathophysiology of disease (27). The current work is the first study to target the anti-TSHR autoimmune process specifically in humans. The treatment, consisting of a mixture of two soluble TSHR ECD peptides, which was administered 10 times to each participant over 18 weeks by i.d. injection, appears to be safe, with mild injection-site swelling and pain as the most frequent AEs. The treatment was also well tolerated, with 10/12 participants finishing the study, despite the requirement for a two-hour in-hospital observation period following each dose. In addition, 7/10 subjects had improvement in their thyroid function over the 18 weeks of ATX-GD-59, with five (50%) normalizing their serum fT3 concentrations, which is the most sensitive serum marker of hyperthyroidism in this context. In concert with reductions in free thyroid hormones, serum TSHR autoantibodies also reduced, and these correlated with improvement in hyperthyroidism. While falling short of a formal demonstration of efficacy in this first-in-human study, these early results suggest that ATX-GD-59 may have a significant disease-modifying therapeutic effect in GD.

During the design of the study, evidence of the potential therapeutic efficacy of ATX-GD-59 was sought, without confounding the assessment of thyroid function by co-administration of ATDs. For this reason, a decision was made to include participants who were relatively mildly affected by hyperthyroidism (inclusion criteria serum fT3 < 15 pmol/L, fT4 < 35 pmol/L) and who might have few symptoms and a low risk of complications from thyrotoxicosis during the study. While this was a successful strategy, in that no patient needed ATD rescue therapy for symptomatic thyrotoxicosis before the final dose of ATX-GD-59, it does mean that these results should be considered as most relevant to GD patients with mild to moderate thyrotoxicosis at the moment. Another key issue is the potential for spontaneous remission of GD. McLarty *et al*. reported spontaneous remission of Graves' hyperthyroidism in 2/21 patients treated with propranolol over a six-month period (28). Subsequently, other studies have observed spontaneous remission in between 10% and 25% of GD patients treated with propranolol alone during three to eight months of treatment (29,30). Thus, the finding of improvement in hyperthyroidism in 7/10 subjects and of normalization of fT3 in five ATX-GD-59-treated subjects is above what would be predicted from spontaneous remission alone. Nevertheless, a formal proof of efficacy study using a placebo-controlled design is needed to confirm this.

GD is one of the commonest autoimmune disorders, and none of the current treatment options are ideal. In particular, ATDs are effective in producing long-term remission in only 50% of patients, and both thyroidectomy and radioiodine therapy lead to long-term medication dependence. Additionally, Graves' orbitopathy affects up to 40% of patients, and current management is unsatisfactory, with a significant minority being left with residual visual impairment, diplopia, or disfiguring changes to the appearance of their eyes. Emerging antigen-specific immunotherapies such as ATX-GD-59 hold several theoretical attractions for treatment of autoimmune, inflammatory, and allergic diseases (15–17,31). As these therapies are directed solely at restoring immune tolerance to the immunodominant epitopes involved in the aberrant autoimmune response, they do not cause generalized immunosuppression with the associated risk of infection, nor do they skew the immune response, putting the patient at risk of different immune-mediated conditions. Notably, cases of immune complex–mediated arthralgia and inflammatory bowel disease have been reported in GD patients following treatment with B-lymphocyte-depleting monoclonal antibodies (32). Furthermore, GD is a particularly attractive model, since if immune tolerance can be restored, the underlying thyroid endocrine function is likely to remain intact for many years, meaning patients have the potential for effective long-term cure. Patients with significant Graves' orbitopathy were excluded from the current study. However, as there is a significant correlation between the development and severity of orbitopathy and TSHR autoantibody concentrations (33,34), it is possible that ATX-GD-59 or a similar TSHR peptide combination could prove efficacious in this most challenging condition. Future studies of ATX-GD-59 in both hyperthyroid GD and Graves' orbitopathy patient cohorts are now required to understand its full therapeutic potential.

## Supplementary Material

Supplemental data
