# Supplementary material for: Antigen-Specific Immunotherapy with Thyrotropin Receptor Peptides in Graves' Hyperthyroidism: A Phase I Study
Source: Thyroid. 2019 Jul 17;29(7):1003–11. doi: 10.1089/thy.2019.0036 (PMC6648194; doi:10.1089/thy.2019.0036)
Supplement: Supplemental data [file Supp_Fig1.pdf]

Supplementary Data

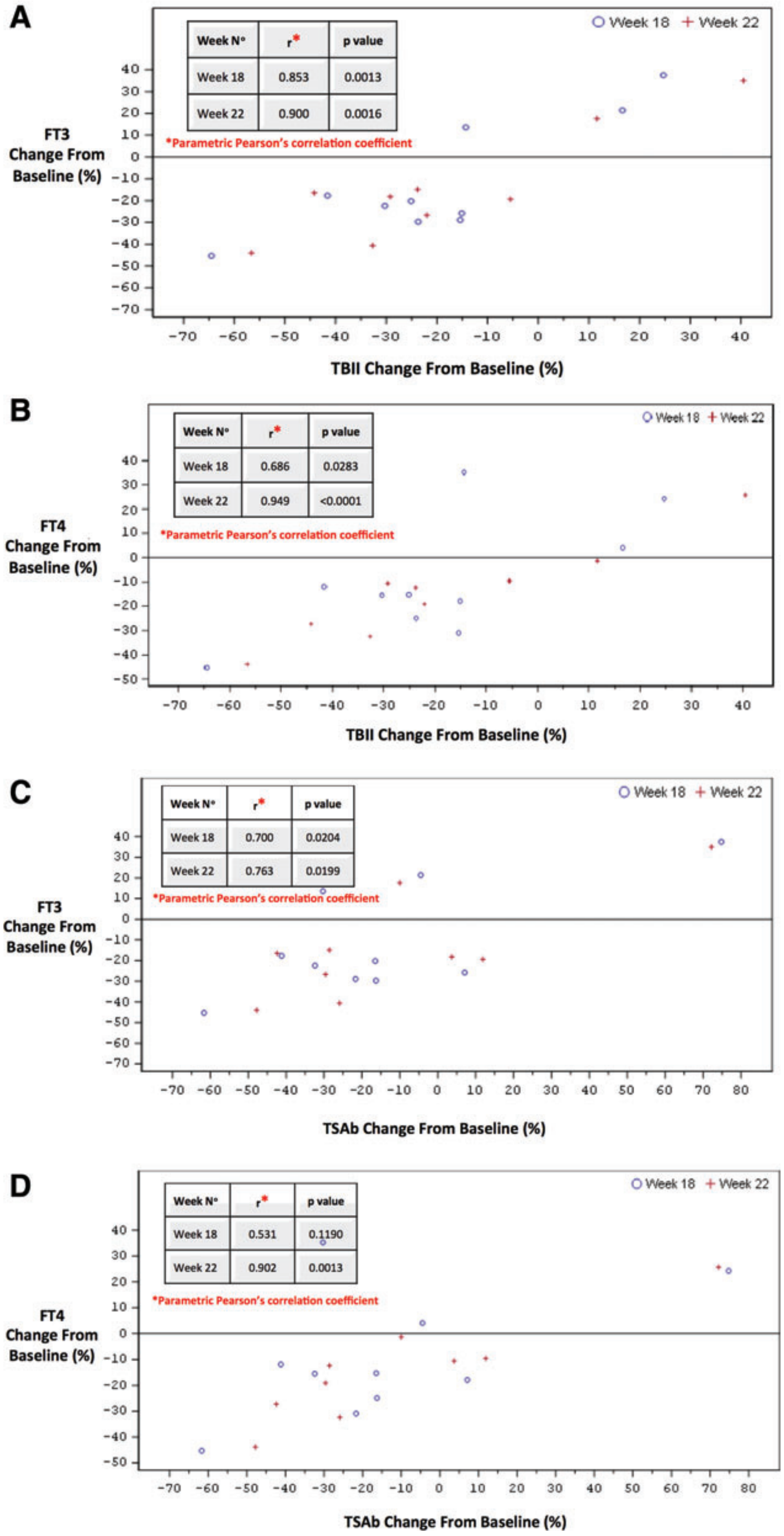

SUPPLEMENTARY FIG. S1. Scatter plot of % change in fT3 (**A**) or fT4 (**B**) compared to % change in TRAb concentrations from baseline at week 18 (blue circle) and week 22 (red cross); and % change in fT3 (**C**) or fT4 (**D**) compared to % change in TSAb concentrations from baseline at week 18 (blue circle) and week 22 (red cross). For both fT3 and fT4 changes, there was a significant correlation with change in TRAb at weeks 18 and 22.
